# Interpretable machine learning model for predicting acute kidney injury in critically ill patients

**DOI:** 10.1186/s12911-024-02537-9

**Published:** 2024-05-31

**Authors:** Xunliang Li, Peng Wang, Yuke Zhu, Wenman Zhao, Haifeng Pan, Deguang Wang

**Affiliations:** 1 Department of Nephrology, The Second Affiliated Hospital of Anhui Medical University, Hefei, China; 2 Teaching Center for Preventive Medicine, School of Public Health, Anhui Medical University, Hefei, China; 3 Department of Epidemiology and Biostatistics, School of Public Health, Anhui Medical University, Hefei, China

**Keywords:** Acute kidney injury, Machine learning, Interpretability, Prediction model, Intensive care unit

## Abstract

**Background:**

This study aimed to create a method for promptly predicting acute kidney injury (AKI) in intensive care patients by applying interpretable, explainable artificial intelligence techniques.

**Methods:**

Population data regarding intensive care patients were derived from the Medical Information Mart for Intensive Care IV database from 2008 to 2019. Machine learning (ML) techniques with six methods were created to construct the predicted models for AKI. The performance of each ML model was evaluated by comparing the areas under the curve (AUC). Local Interpretable Model-Agnostic Explanations (LIME) method and Shapley Additive exPlanation values were used to decipher the best model.

**Results:**

According to inclusion and exclusion criteria, 53,150 severely sick individuals were included in the present study, of which 42,520 (80%) were assigned to the training group, and 10,630 (20%) were allocated to the validation group. Compared to the other five ML models, the eXtreme Gradient Boosting (XGBoost) model greatly predicted AKI following ICU admission, with an AUC of 0.816. The top four contributing variables of the XGBoost model were SOFA score, weight, mechanical ventilation, and the Simplified Acute Physiology Score II. An AKI and Non-AKI cases were predicted separately using the LIME algorithm.

**Conclusion:**

Overall, the constructed clinical feature-based ML models are excellent in predicting AKI in intensive care patients. It would be constructive for physicians to provide early support and timely intervention measures to intensive care patients at risk of AKI.

**Supplementary Information:**

The online version contains supplementary material available at 10.1186/s12911-024-02537-9.

## Introduction

Acute kidney injury (AKI) is the most severe, common, and life-threatening complication in hospitalized patients and is associated with high morbidity and mortality rates [1]. It has been demonstrated that AKI affects approximately 30–60% of critically ill patients, especially those in the intensive care unit (ICU) [1]. Despite the recent advances in clinical care and dialysis technology, the occurrence of AKI in ICU patients has a mortality rate of up to 50%, which is 1.5 to 2-fold to that of ICU patients without AKI [2, 3]. However, if detected and managed promptly, interventions guided by established recommendations, such as those provided by KDIGO, may mitigate the risk of further deterioration in AKI patients [4]. Therefore, identifying individuals at high risk of AKI is vital for managing critically ill patients.

Artificial intelligence (AI) and machine learning (ML) represent emerging technologies that could use large amounts of health-related data to help physicians make better clinical decisions and improve individual health outcomes. While serum creatinine (Scr) and urine output serve as diagnostic criteria for AKI, delays in their detection may occur. Therefore, early identification of patients at risk of developing AKI is crucial to create a window for preventive interventions and mitigate the risk of further deterioration. Several previous studies have developed various ML-based models to predict AKI in critically ill patients due to the potential benefits of early detection of AKI [5, 6]. It is critical to remove the mystery surrounding ML since doing so makes it simpler for doctors to comprehend the reasoning behind ML [7]. In order to explain why ML makes the choices it does, a new field called Explainable AI (XAI) has emerged. Two of the most popular methods for explaining are Local Interpretable Model-Agnostic Explanation (LIME) and Shapley Additive ExPlanation (SHAP) [8, 9]. Novel interpretable approaches have been effectively utilized to explain ML models for preventing hypoxemia during surgery [10], predicting mortality in sepsis and AKI [9, 11], predicting the occurrence of AKI following cardiac surgery [12], and predicting antibiotic resistance [13].

To the best of our knowledge, the reliability and robustness of explanatory techniques for detecting AKI in critically sick patients have rarely been studied. Therefore, the present study was conducted to construct an ML approach for the early prediction of AKI in ICU patients and to apply XAIs to make ML more transparent and interpretable.

## Methods

### Data sources

The relevant data were retrieved from the Medical Information Mart Database for Intensive Care IV (MIMIC IV), which includes the anonymized medical records of 76,540 patients hospitalized in the ICU at Beth Israel Deaconess Medical Center between 2008 and 2019 [14]. MIMIC IV was set up with the approval of the Institutional Review Board at the Massachusetts Institute of Technology. All participant data were anonymized to safeguard their privacy. Due to using anonymized health records, ethical approval and informed consent were not required. This study adheres to the ethical criteria outlined in the Helsinki Declaration of 1964. The author XL passed the National Institutes of Health’s exam on protecting the privacy of human research participants (certification number 35,970,146) to gain entry to the database.

### Study population

All adult (aged 18 years old and older) patients who were admitted to the ICU from the MIMIC IV database were included in this study. If a patient was recently admitted to the ICU more than once, we only considered the first admission.

### Data collection

Baseline characteristics, including demographic information, comorbidities, vital signs, laboratory results, medical interventions, disease severity scores, etc., were carefully reviewed and collected. The definitions of comorbidities were followed with the Implementation of the International Statistical Classification of Disease and Related Health Problems, 10th Revision coding systems recorded by hospital staff at the time of patient discharge [15], including congestive heart failure, peptic ulcer disease, myocardial infarction, peripheral vascular disease, diabetes, dementia, chronic pulmonary disease, rheumatic disease, cerebrovascular disease, cancer, paraplegia, liver disease, renal disease, and acquired immune deficiency syndrome. Severe organ failure due to ineffective immune response to infection was identified as sepsis. During the first 24 h when the patient was admitted to the ICU, the average values of the patient’s vital signs (heart rate, mean arterial pressure, respiration rate, body temperature, and SpO_2_) were measured,, and the highest value of the biochemical laboratory tests (hematocrit, hemoglobin, platelets, white blood cell, blood urea nitrogen, anion gap, international normalized ratio, Scr, serum glucose, serum calcium, serum chloride, bicarbonate, serum potassium, serum sodium) were also determined. The baseline of serum Scr level was utilized to calculate the estimated glomerular filtration rate (eGFR). Medical interventions included dialysis, vasopressors, and mechanical ventilation during the first 24 h after ICU admission. Within the first 24 h following the patient’s admission to the ICU, we determined the initial value for the Sequential Organ Failure Assessment (SOFA) score and the Simplified Acute Physiology Score II (SAPS II) score, which measures the severity of an illness.

### Definition of AKI

The Kidney Disease: Improving Global Outcomes 2012 guidelines were used to diagnose AKI during hospitalization in an ICU [16]: increase in Scr of at least 1.5-fold from baseline within the previous seven days; increase in Scr of at least 0.3 mg/dl within the previous 48 h, or urinary output of at least 0.5 ml/kg per hour for 6 h or more. The patient’s urine output was measured hourly after admission. We used the lowest Scr value seven days before the patient was admitted to the ICU as the baseline Scr level. When pre-ICU Scr was unavailable, the first Scr value recorded after admission to the ICU was utilized as the baseline Scr. In this study, 43,317 (81.5%) patients had Scr data within seven days prior to admission to the ICU.

### Management of missing data

In the MIMIC IV database, missing data is a widespread problem that needs to be addressed. Less than 20% of all variables in this study were missing (Supplementary Table S1). Multiple imputation methods were used to recreate the missing variables.

### Statistical analysis

Python (Version 3.9.12) and R (Release 4.2.1, Foundation R for Statistical Computing) were used for all statistical analyses. Two-tailed *P* < 0.05 was set as the statistical significance. Due to the skewed distribution, the median and interquartile ranges were used to describe continuous data, and the Wilcoxon rank-sum test was used to draw comparisons between groups. Chi-square tests or Fisher’s exact probability approach was used to compare categorical variables’ numerical and percentage values.

### ML models

The data were randomly split into two proportions: the training (80%) and validation (20%) sets. The supervised ML with logistic regression, support vector machine (SVM), k nearest neighbour (KNN), decision tree, random forest (RF), and extreme gradient boosting (XGBoost) methods were applied to construct the predictive models. In logistic regression, we used a variance inflation factor (VIF) to assess collinearity among predictors and restricted predictors with VIF values less than 5 to be used in subsequent model construction. Each ML algorithm’s default hyper-parameters were implemented to establish the model. Ten-fold cross-validation was applied on the training and validation data to avoid overfitting to find the best settings for the hyperparameters. In brief, ten roughly equal-sized subgroups were drawn randomly from the training set, of which nine were put into the model, while the remaining one was utilized for model validation. In order to ensure that each subset could be used as a validation set, we repeated this procedure ten times. The area under the curve (AUC), F1 score, precision and recall were calculated for each model. The best model of each method was selected when it showed the largest AUC. We also performed decision curve analysis (DCA) and plotted calibration curves to compare each model’s predictive power and clinical usefulness. We used SHAP values to display major factors impacting AKI risk to examine the significance of individual characteristics affecting model output. The LIME algorithm was then applied to make the model’s predictions.

## Results

### Baseline characteristics

**Fig. 1:**
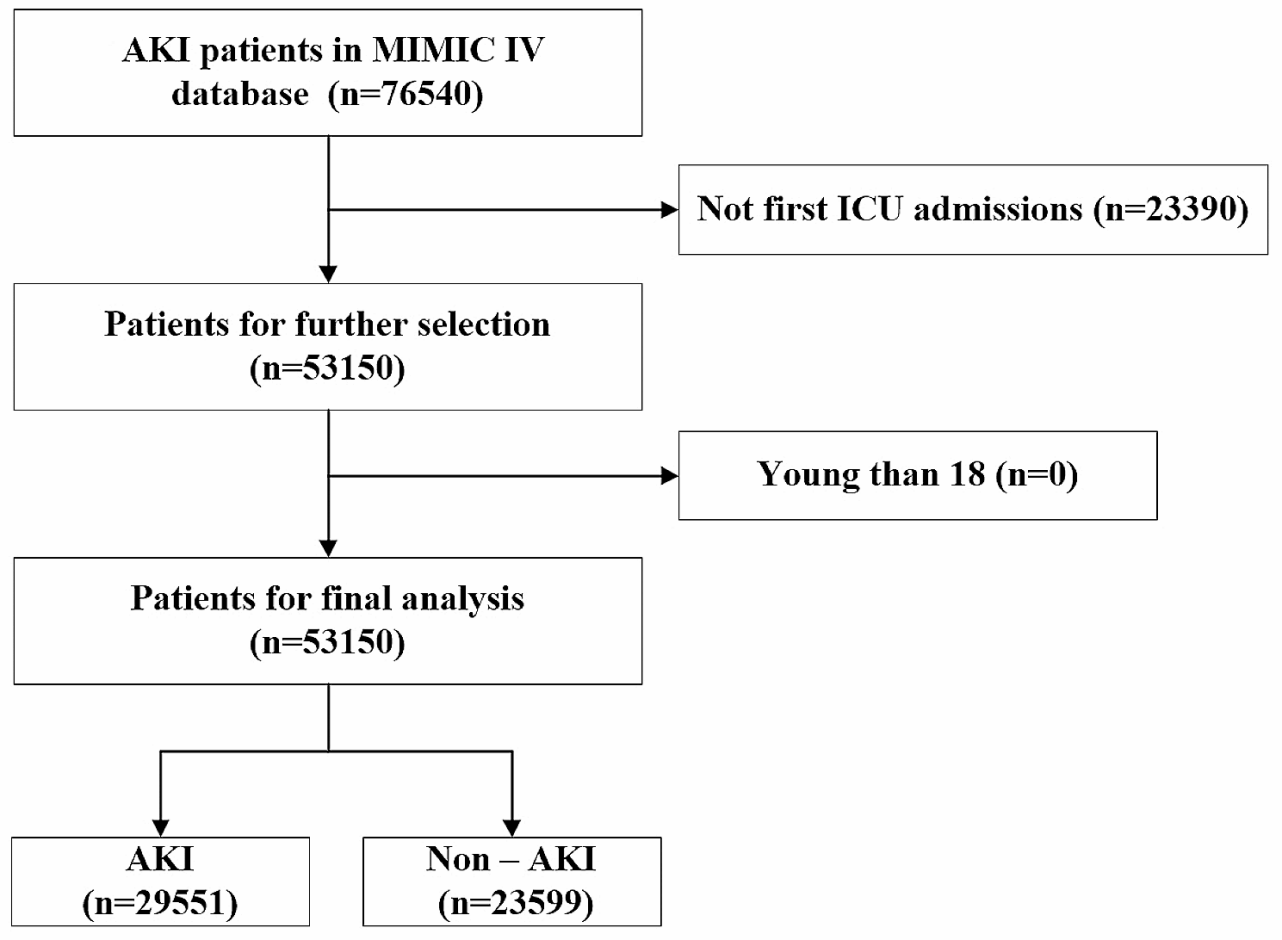
Flow chart of patient selection from the MIMIC IV database. Abbreviations: MIMIC IV: Medical Information Mort for Intensive Care IV, ICU: intensive care unit, AKI: acute kidney injury

After carefully reviewing the MIMIC IV database, we found 76,540 records of ICU admissions reports. According to the exclusion criteria, we excluded multiple ICU admissions for the same patients, and there were 53,150 patients included, where 29,551 patients were diagnosed with AKI (Fig. 1).

Table 1 shows the differences in baseline characteristics between AKI and non-AKI groups. Patients with AKI had increased levels of age, body weight, SOFA and SAPS II values than non-AKI cases; the hospitalized male patients were more likely to develop AKI than female patients. Compared to non-AKI patients, AKI patients had more complications, including congestive heart failure, myocardial infarction, peripheral vascular disease, diabetes, dementia, chronic pulmonary disease, rheumatic disease, cancer, liver disease, renal disease and sepsis. Moreover, there were significant differences in most vital signs and laboratory data between AKI and non-AKI groups, with most parameters related to increased illness severity within the AKI group. MAP was lower in AKI patients than in non-AKI patients. Patients with AKI were also more likely to undergo dialysis, vasopressors, and mechanical ventilation during the first 24 h after ICU admission.

**Table 1:** Demographic and clinical characteristics of study population at baseline

Variables	Total (*n* = 53,150)	Non –AKI (*n* = 23,599)	AKI (*n* = 29,551)	*P* value
**Age (years)**	66.76 [54.49, 78.24]	63.27 [50.16, 75.74]	69.12 [57.90, 79.75]	< 0.001
**Sex, male, n (%)**	29,797 (56.1)	12,832 (54.4)	16,965 (57.4)	< 0.001
**Weight (kg)**	78.45 [65.90, 93.10]	75.00 [63.10, 88.00]	81.50 [68.60, 97.10]	< 0.001
**Ethnicity, n (%)**				< 0.001
White	35,668 (67.1)	15,676 (66.4)	19,992 (67.7)	
Black	4874 (9.2)	2349 (10.0)	2525 (8.5)	
Other	12,608 (23.7)	5574 (23.6)	7034 (23.8)	
**Congestive heart failure, n (%)**	12,622 (23.7)	3769 (16.0)	8853 (30.0)	< 0.001
**Peptic ulcer disease, n (%)**	1457 (2.7)	724 (3.1)	733 (2.5)	< 0.001
**Myocardial infarction, n (%)**	8531 (16.1)	2951 (12.5)	5580 (18.9)	< 0.001
**Peripheral vascular disease, n (%)**	5820 (11.0)	2036 (8.6)	3784 (12.8)	< 0.001
**Diabetes, n (%)**	14,613 (27.5)	5386 (22.8)	9227 (31.2)	< 0.001
**Dementia, n (%)**	1930 (3.6)	778 (3.3)	1152 (3.9)	< 0.001
**Chronic pulmonary disease, n (%)**	12,398 (23.3)	4879 (20.7)	7519 (25.4)	< 0.001
**Rheumatic disease, n (%)**	1717 (3.2)	684 (2.9)	1033 (3.5)	< 0.001
**Cerebrovascular disease, n (%)**	8539 (16.1)	3831 (16.2)	4708 (15.9)	0.352
**Cancer, n (%)**	7723 (14.5)	3426 (14.5)	4297 (14.5)	0.949
**Paraplegia, n (%)**	2748 (5.2)	1175 (5.0)	1573 (5.3)	0.078
**Liver disease, n (%)**	5766 (10.8)	2017 (8.5)	3749 (12.7)	< 0.001
**Renal disease, n (%)**	9386 (17.7)	2837 (12.0)	6549 (22.2)	< 0.001
**AIDS, n (%)**	284 (0.5)	158 (0.7)	126 (0.4)	< 0.001
**Sepsis, n (%)**	23,901 (45.0)	6684 (28.3)	17,217 (58.3)	< 0.001
**Heart rate (beats/minute)**	82.83 [73.25, 94.16]	81.83 [72.08, 93.00]	83.61 [74.28, 95.14]	< 0.001
**MAP (mmHg)**	77.52 [71.27, 85.61]	79.86 [72.87, 88.00]	75.86 [70.27, 83.30]	< 0.001
**Respiratory rate (beats/minute)**	18.38 [16.39, 20.96]	18.06 [16.20, 20.42]	18.66 [16.57, 21.42]	< 0.001
**Body temperature (°C)**	36.81 [36.59, 37.07]	36.81 [36.61, 37.04]	36.81 [36.56, 37.10]	0.878
**SpO**_**2**_ **(%)**	97.07 [95.69, 98.36]	96.92 [95.62, 98.19]	97.19 [95.76, 98.48]	< 0.001
**Hematocrit (%)**	35.20 [31.00, 39.60]	35.80 [31.40, 40.10]	34.70 [30.70, 39.20]	< 0.001
**Hemoglobin (g/dL)**	11.70 [10.20, 13.20]	11.90 [10.30, 13.40]	11.40 [10.00, 13.00]	< 0.001
**Platelets (K/uL)**	210.00 [158.00, 275.00]	218.00 [167.00, 279.00]	204.00 [152.00, 272.00]	< 0.001
**WBC (K/uL)**	12.30 [8.80, 16.70]	11.00 [8.00, 15.00]	13.30 [9.70, 18.00]	< 0.001
**BUN (mg/dL)**	19.00 [14.00, 30.00]	17.00 [12.00, 24.00]	22.00 [15.00, 35.00]	< 0.001
**Anion gap (mEq/L)**	15.00 [13.00, 18.00]	15.00 [13.00, 17.00]	16.00 [13.00, 19.00]	< 0.001
**INR**	1.30 [1.10, 1.50]	1.20 [1.10, 1.40]	1.30 [1.20, 1.60]	< 0.001
**Serum creatinine (mg/dL)**	1.00 [0.80, 1.40]	0.90 [0.70, 1.20]	1.10 [0.80, 1.70]	< 0.001
**Serum glucose (mg/dL)**	137.00 [113.00, 178.00]	131.00 [109.00, 166.00]	143.00 [117.00, 187.00]	< 0.001
**Serum calcium, (mg/dL)**	8.60 [8.10, 9.00]	8.60 [8.20, 9.10]	8.50 [8.10, 9.00]	< 0.001
**Serum chloride, (mEq/l)**	106.00 [102.00, 109.00]	106.00 [102.00, 109.00]	106.00 [102.00, 110.00]	< 0.001
**Bicarbonate (mmol/L)**	24.00 [22.00, 27.00]	24.00 [22.00, 27.00]	24.00 [22.00, 27.00]	< 0.001
**Serum potassium (mEq/L)**	4.40 [4.00, 4.80]	4.20 [3.90, 4.60]	4.50 [4.10, 5.00]	< 0.001
**Serum sodium (mEq/L)**	140.00 [137.00, 142.00]	140.00 [137.00, 142.00]	140.00 [137.00, 142.00]	0.372
**PT (s)**	14.00 [12.40, 16.60]	13.30 [12.00, 15.50]	14.60 [12.80, 17.50]	< 0.001
**PTT (s)**	31.30 [27.50, 40.00]	30.10 [26.90, 36.20]	32.60 [28.15, 44.10]	< 0.001
**eGFR, ml/min/1.73 m** ^**2**^	101 [81, 106]	101 [82, 107]	101 [81, 105]	< 0.001
**Dialysis, n (%)**	1668 (3.1)	167 (0.7)	1501 (5.1)	< 0.001
**Vasopressors use, n (%)**	2004 (3.8)	258 (1.1)	1746 (5.9)	< 0.001
**Mechanical ventilation, n (%)**	38,366 (72.2)	13,281 (56.3)	25,085 (84.9)	< 0.001
**SOFA score**	4.00 [2.00, 6.00]	2.00 [1.00, 4.00]	5.00 [3.00, 8.00]	< 0.001
**SAPS II score**	33.00 [25.00, 42.00]	28.00 [20.00, 36.00]	37.00 [29.00, 47.00]	< 0.001

Abbreviations: AKI: acute kidney injury, AIDS: acquired immune deficiency syndrome, MAP: mean arterial pressure, SpO_2_: oxygen saturation, WBC: white blood cell, BUN: blood urea nitrogen, INR: international normalized ratio, PT: prothrombin time, PTT: partial thromboplastin time, eGFR: estimated glomerular filtration rate, SOFA: sequential organ failure assessment, SAPS II: Simplified Acute Physiology Score II

### Model construction and validation

**Fig. 2:**
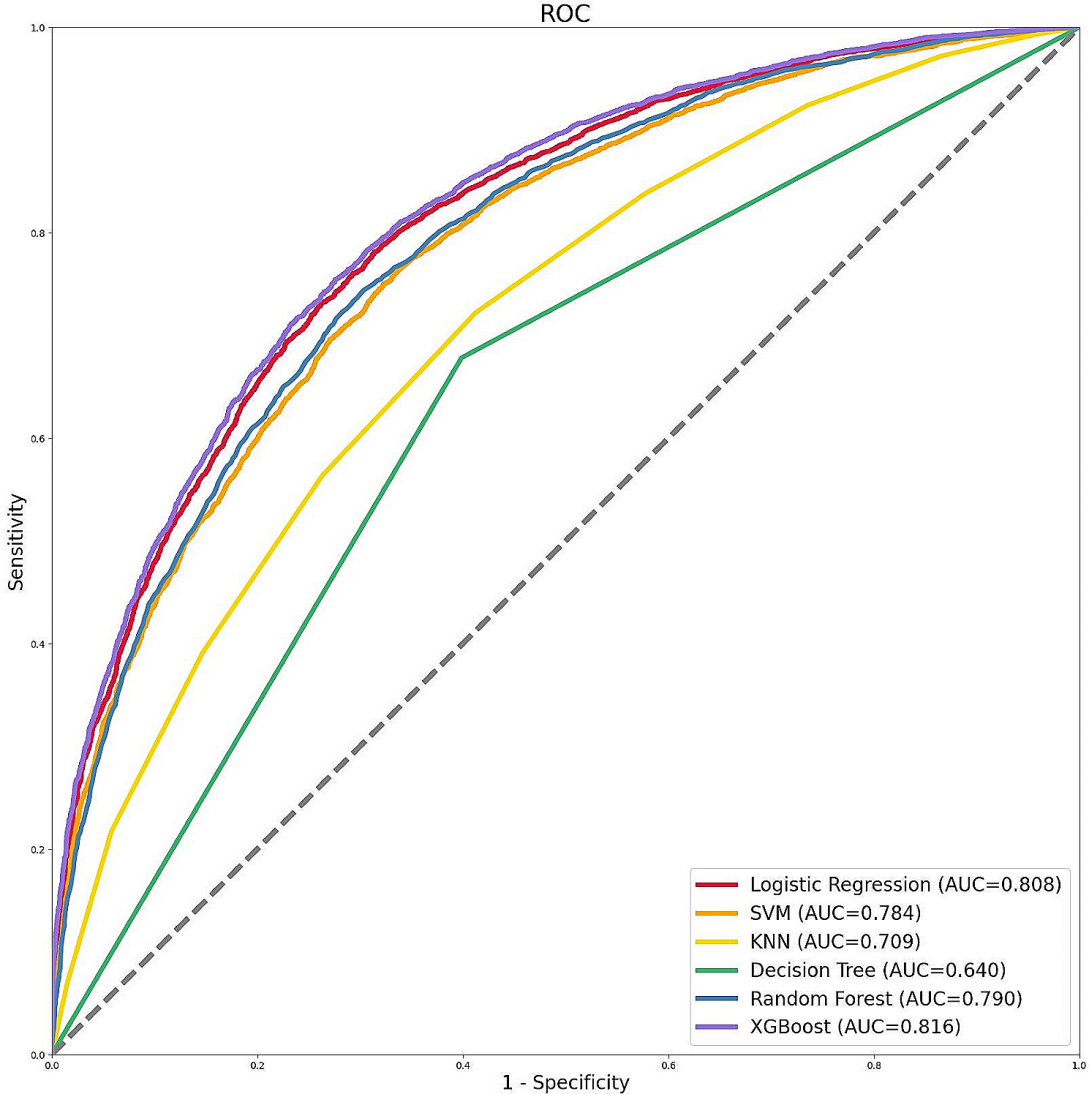
ROC curve of the six models. Abbreviations: ROC: receiver operating characteristic, SVM: support vector machine, KNN, k-nearest neighbors, AUC: area under the curve

The number of patients was 42,520 and 10,630 in the training and validation datasets, respectively. There were no significant differences in the baseline features between the training and validation sets (Supplementary Table S2). We applied ML approaches to predict AKI throughout the hospitalization period after ICU admission using six methods, including logistic regression, SVM, KNN, decision tree, RF, and XGBoost. The discriminative abilities of the ROC curve among six models are displayed in Fig. 2. The XGBoost model showed the highest AUC for predicting AKI following ICU admission (AUC = 0.816), followed by the logistic regression model (AUC = 0.808), the RF model (AUC = 0.790), the SVM model (AUC = 0.784), the KNN model (AUC = 0.709), and the decision tree model (AUC = 0.640). The respective performance measures among the six models are listed in Table 2. Regarding discriminating ability, the XGBoost model had considerable values, with an accuracy of 0.743%, an F1 score of 0.774, and a recall of 0.794. The DCA and calibration curves showed that the XGBoost model had the best predictive power and clinical utility among the six models (Supplementary Figure S1 and Supplementary Figure S2).

**Table 2:** The performance comparisons of the ML models in the testing set

Models	AUC (95% CI)	Accuracy	Recall	F1 score
Logistic regression	0.808 (0.800–0.816)	0.738	0.783	0.769
Support vector machine	0.784 (0.776–0.793)	0.718	0.771	0.753
k-Nearest neighbor	0.709 (0.700–0.719)	0.662	0.722	0.704
Decision tree	0.640 (0.630–0.650)	0.718	0.674	0.678
Random forest	0.790 (0.781–0.798)	0.720	0.800	0.760
XGBoost	0.816 (0.808–0.824)	0.743	0.794	0.774

Abbreviations: AUC: area under the curve, CI: confidence interval, XGBoost: Extreme Gradient Boosting

### Model explainability

**Fig. 3:**
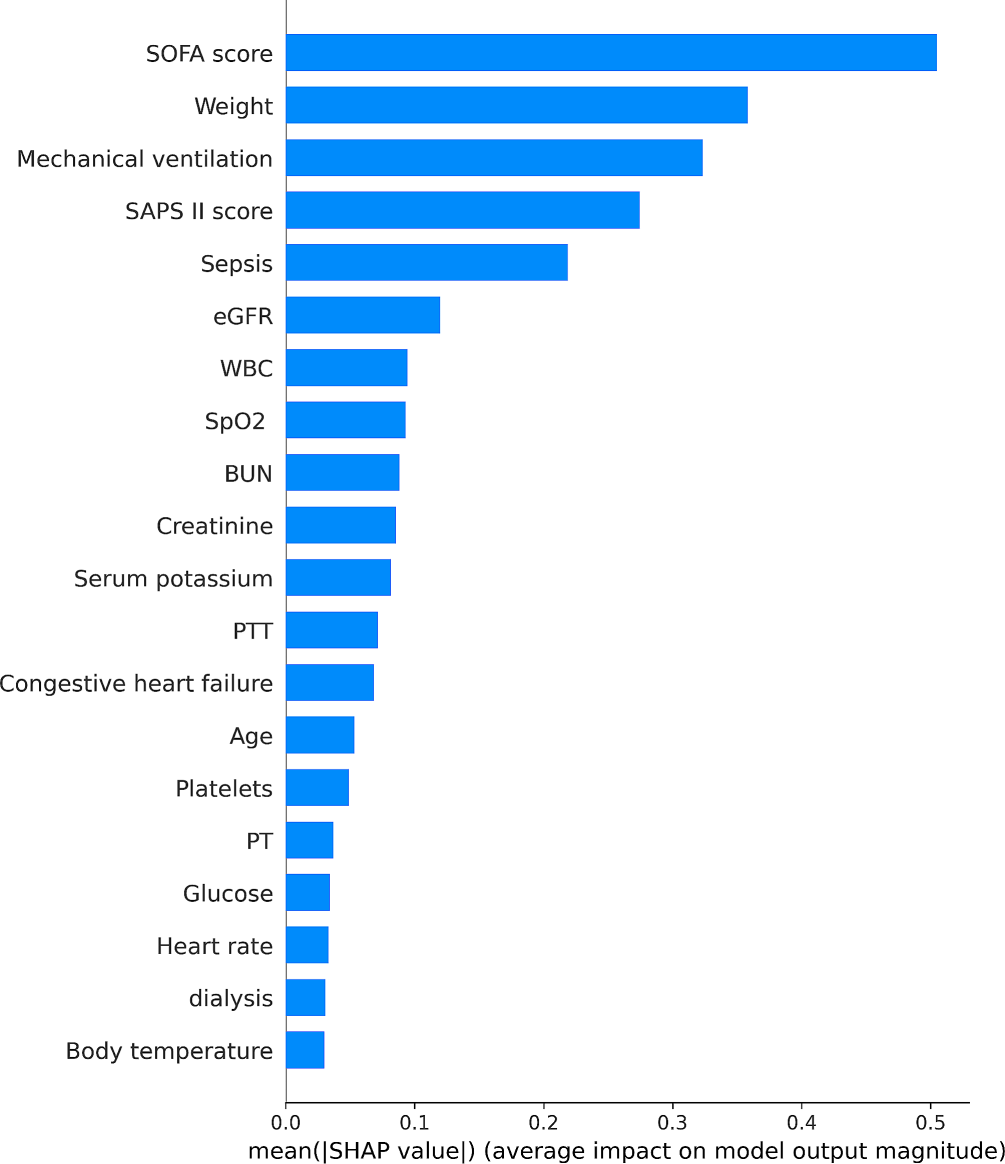
The top 20 important features derived from the XGBoost model. The higher the SHAP value of a feature, the higher the probability of acute kidney injury development. Each line represents a feature, and the abscissa is the SHAP value. Red dots represent higher feature values, and blue dots represent lower feature values.Abbreviations: SHAP: Shapley Additive explanation, SOFA: sequential organ failure assessment, SAPS II: Simplified Acute Physiology Score II, eGFR: estimated glomerular filtration rate, WBC: white blood cell, SpO2: oxygen saturation, BUN: blood urea nitrogen, PTT: partial thromboplastin time, PT: prothrombin time

**Fig. 4:**
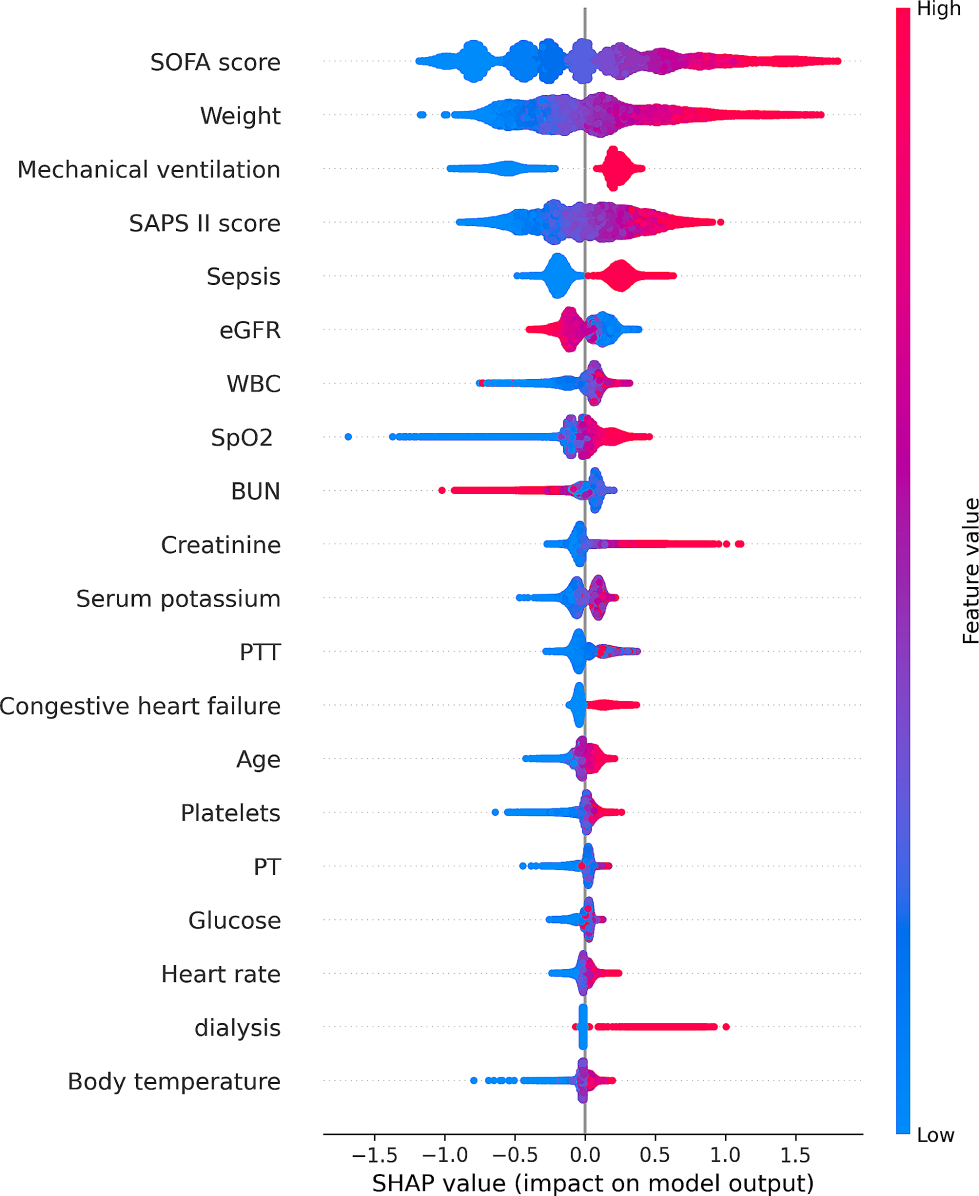
SHAP summary plot of the top 20 features of the XGBoost model. Ranking of feature importance indicated by SHAP. The matrix plot depicts the importance of each covariate in the development of the final predictive model. Abbreviations: SHAP: Shapley Additive explanation, SOFA: sequential organ failure assessment, SAPS II: Simplified Acute Physiology Score II, eGFR: estimated glomerular filtration rate, WBC: white blood cell, SpO2: oxygen saturation, BUN: blood urea nitrogen, PTT: partial thromboplastin time, PT: prothrombin time

**Fig. 5:**
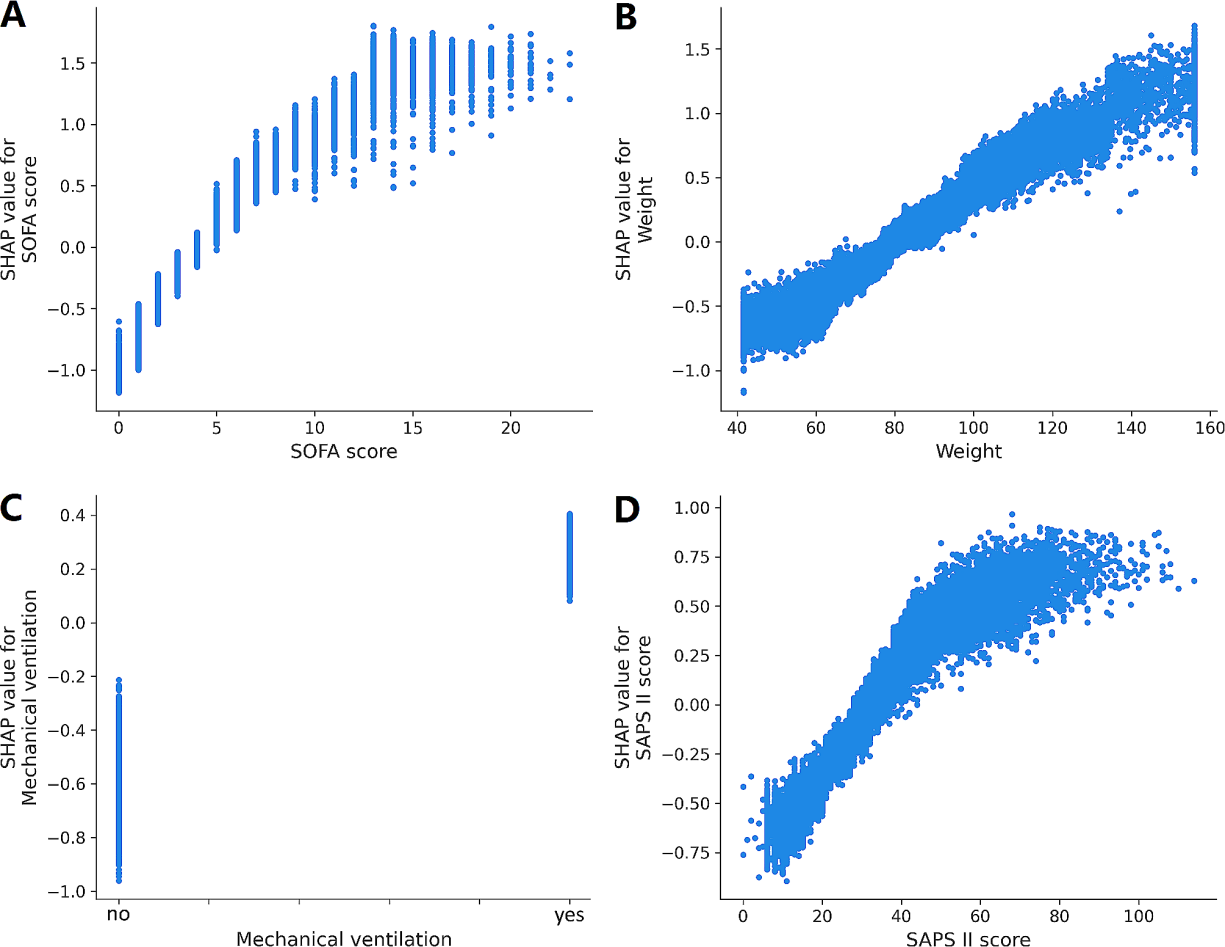
SHAP dependence plot of the XGBoost model. (**A**) SOFA score; (**B**) Weight; (**C**) Mechanical ventilation; (**D**) SAPS II score. SHAP values for specific features exceed zero, representing an increased risk of acute kidney injury development. Abbreviations: SHAP: Shapley Additive explanation, SOFA: sequential organ failure assessment, SAPS II: Simplified Acute Physiology Score II

In order to unveil the significant contributor to the prediction model, we plotted the SHAP summary of XGBoost and the top 20 features of the prediction model. For the XGBoost model, the SHAP summary graphic (Fig. 3) ranked the features in the significance order. The top four contributors were the SOFA score, body weight, mechanical ventilation and the SAPS II score. Additionally, we utilized SHAP’s dependency analysis to visually display a single input’s impact on the XGBoost prediction model’s final result (Fig. 4). A SHAP value of more than zero indicates an increased risk of developing AKI. Figure 5 provided more details on the top four contributors of the prediction model XGBoost.

**Fig. 6:**
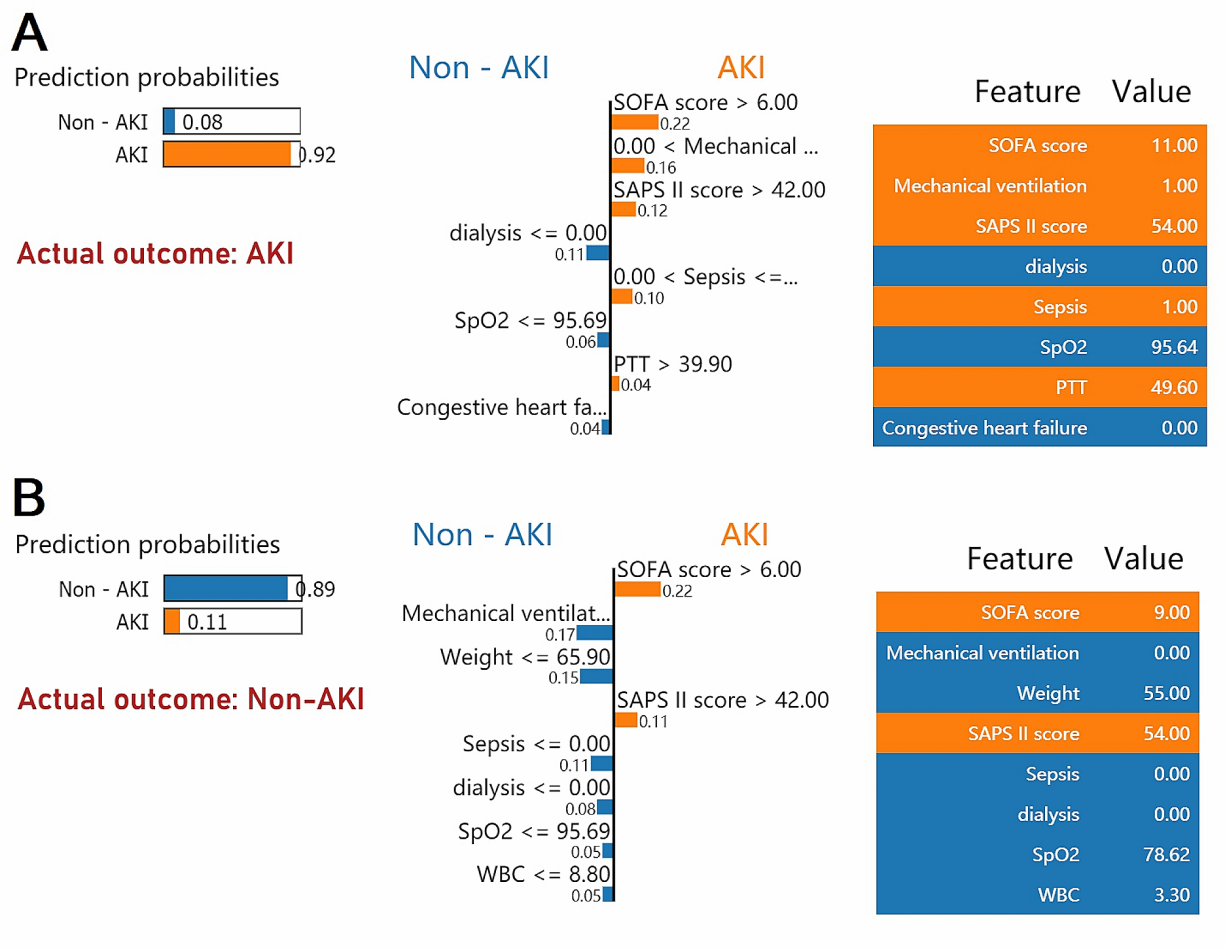
LIME algorithm for explaining individual’s prediction results. Screenshot of the AKI prediction in patients. (**A**) present an AKI case with the LIME algorithm. (**B**) present a Non-AKI case with the LIME algorithm. The left part of the figure shows predicted results using LIME. The middle part presents the top 8 variables that had the greatest impact on survival or death from top to bottom. The length of the bar for each feature indicates the importance (weight) of that feature in making the prediction. A longer bar indicates a feature that contributes more to survival or death. The right panel shows the critical values of these 8 variables when they had the greatest impact on survival or death. Abbreviations: LIME: Local Interpretable Model-Agnostic Explanations, AKI: acute kidney injury, SOFA: sequential organ failure assessment, SAPS II: Simplified Acute Physiology Score II, SpO2: oxygen saturation, PTT: partial thromboplastin time, WBC: white blood cell.

The LIME algorithm was then applied to explain the reliability and evaluate the prediction ability of the ML model. Two random samples were selected from the validation for an individual’s AKI prediction. A case of AKI using the LIME algorithm is shown in Fig. 6A. The predicted AKI probability by the XGBoost model was 92%, and it found that a SOFA score of 11, the presence of sepsis, a SAPS II score of 54, the necessity for mechanical ventilation, and a partial thromboplastin time (PTT) of 49.6s were associated with increased risk of AKI. In contrast, the absence of dialysis within the first 24 h of ICU admission and the absence of a history of congestive heart failure were observed to be associated with a lower risk of developing AKI. The patient’s actual outcome was consistent with the XGBoost model’s prediction of AKI. Similarly, Fig. 6B presents a non-AKI case using the LIME algorithm. The probability predicted for AKI by the XGBoost model was 11%. The SOFA score of 9 and SAPS II score of 54 contribute to an increased risk of AKI, while the SpO2 of 78.62% and WBC 3.3 K/ul contributed to a decreased risk of AKI. For this patient, the predicted outcome from the XGBoost model was non-AKI, consistent with the actual outcome of non-AKI.

## Discussion

In the present study, we implemented the ML approaches to construct six ML models with the use of a total of forty-six demographic and clinical variables, in order to assess the likelihood of the development of AKI in critically ill patients. Among the six ML models, it showed that the XGBoost model had the relative great performance on the prediction and discrimination of AKI in critically ill patients. The significance of the characteristics and the impact of demographic and clinical variables on XGBoost’s prediction were also revealed by SHAP values. The XGBoost model was built using several variables, the top-four contributing factors: the SOFA score, weight, mechanical ventilation and the SAPS II score. In addition, the LIME method was utilized to explain the reliability and evaluate the prediction ability of ML model, and it proved that the constructed XGBoost model had a considerable value on the prediction and discrimination of AKI from the critically ill patients.

In our study, the AUC between the XGBoost and Logistic regression models are comparable. However, the practical effectiveness of XGBoost in predicting AKI for critically ill patients is evident in several aspects. XGBoost excels in capturing complex, nonlinear relationships within the dataset, a vital consideration given the intricate nature of critically ill patients. Additionally, the model’s interpretability is enhanced through the use of SHAP values and the LIME algorithm, providing insights into influential factors. The robustness of XGBoost across diverse datasets and its potential for better generalization further contribute to its practical superiority. Moreover, a comprehensive evaluation considering metrics beyond AUC, such as accuracy, recall, and F1 score, consistently demonstrates the favorable performance of XGBoost. Although the AUC values of XGBoost and logistic regression were comparable, the subtle advantages of XGBoost collectively support its validity in predicting AKI, highlighting the clinical relevance of our findings.

Our study revealed that the SOFA score, body weight, mechanical ventilation and the SAPS II score represented the top four factors contributing to the risk of AKI in critically ill patients. Organ dysfunction and disease severity are often measured using SOFA scores [17]. Several studies have demonstrated that a greater SOFA score is strongly associated with an increased likelihood of AKI [18]. However, those factors were not incorporated into the previous ML models when predicting AKI risk in critically ill patients [5, 6]. Among the top four factors, the XGBoost model showed that SOFA score was the most important predictor for AKI. In addition, our study revealed that body weight was closely related to morbidity in AKI. Being overweight increases the likelihood of being obese, which in turn increases the risk of developing AKI by increasing the possibility of glomerular hyperperfusion and hyperfiltration, the hemodynamic and metabolic burden on a single glomerulus, and the activation of adipocyte inflammation and oxidative stress [19]. Additionally, we discovered that mechanical ventilation was strongly linked to AKI in ICU patients. Clinicians commonly use positive-pressure mechanical ventilation to increase ventilation and oxygen saturation in critically ill patients while protecting their airways. Nevertheless, mechanical ventilation has been suggested to have potential long-term harmful effects on the kidneys [20]. The following reasons might explain this. First, mechanical ventilation with positive pressure may influence renal perfusion by raising intrathoracic pressure and decreasing venous return and cardiac output. Second, the renin-angiotensin system may be impacted by mechanical ventilation, leading to decreased renal blood flow. Third, mechanical ventilation may produce a series of inflammatory reactions, which may also lead to AKI. SAPS II score is a commonly used scoring system in the ICU to evaluate the severity of a patient’s disease, and some studies have revealed the positive associations of SAPS II score with AKI risk in postoperative cardiac and septic patients [21, 22], we found that the SAPS II score was another key predictor for AKI, where an increase of SAPS II score was associated with an elevated risk of AKI in critically sick patients. Taken together, the constructed XGBoost model using SOFA score, body weight, mechanical ventilation and the SAPS II score could provide considerable values in predicting AKI in critically ill patients.

This study demonstrates the potential benefits of employing ML models, particularly the XGBoost model, in predicting AKI among critically ill patients in ICUs. While traditional severity of disease scoring systems such as SOFA score, APACHE II, and SAPS II are effective, they often rely on a predefined set of variables and may not capture the full complexity of individual patient profiles. In contrast, our ML models utilize a broader array of clinical features, allowing for a more nuanced and individualized prediction of AKI risk. The superior performance of the XGBoost model, with an AUC of 0.816, highlights its ability to consider a diverse set of features, including weight and mechanical ventilation, which may not be explicitly accounted for in traditional scoring systems. By providing clinicians with additional insights into AKI risk, our models facilitate early identification and intervention, ultimately enhancing patient care in the ICU setting.

The timely and accurate prediction of AKI in critically ill patients is crucial for identifying patients at high risk of clinical deterioration and taking preventative interventions promptly, and it would be helpful for the reduction of morbidity and mortality of AKI in those patients with critically ill. A growing body of literature has highlighted the considerable value of ML approaches in predicting AKI in critically ill patients. Chiofolo and colleagues created an ML model for predicting AKI using an autonomous continuous random forest algorithm, which can identify the possible high-risk AKI individuals in ICU patients [5]. At the same time, Le et al. built an ML model to predict AKI in critically ill patients using convolutional neural networks, which better predicted AKI than the traditional SOFA scoring system [6]. However, previous ML models were developed using insufficient algorithm resources and could not describe how they worked clearly. In the current study, we compared and contrasted several different ML methods, including the LR, SVM, XGBoost, KNN, Decision tree, and RF, to determine the ML models with the best discrimination and accuracy. We found that the XGBoost model yielded the best results; further SHAP values and the LIME technique allowed us to understand the primary factors influencing the model’s prediction ability and improve the interpretability of the XGBoost model.

The application of interpretable machine learning models, notably the XGBoost model, for predicting AKI in intensive care patients holds significant clinical relevance. Early identification of AKI is crucial for timely intervention and improved patient outcomes. The transparency and interpretability of our models, achieved through techniques like LIME, enhance their usability for clinicians. The identification of key contributing variables, including SOFA score, weight, mechanical ventilation, and the SAPS II, provides valuable insights into factors associated with AKI in critically ill patients. Moving forward, translating these findings into clinical practice requires further validation in diverse settings and populations. Collaboration between researchers, clinicians, and healthcare institutions is essential for developing user-friendly interfaces and decision support tools. Prospective studies are needed to evaluate the real-world impact on clinical decision-making and patient outcomes. Additionally, education and training initiatives are crucial to ensure healthcare providers can effectively interpret and integrate these models into their workflows. In summary, while our study lays the foundation for AKI prediction, ongoing efforts are necessary to bridge the gap between research findings and tangible clinical benefits.

Nevertheless, in the current study, several shortcomings should be noticed. First, the current study was limited in concluding causation because it was a retrospective modelling study conducted at a single centre utilizing the MIMIC IV database. Second, we estimated specific missing data using the fill method, which could result in a discrepancy with the valid number. Third, using the lowest Scr value from the seven days prior to the patient’s ICU admission as the baseline Scr level may be biased. This method may not accurately reflect true baseline renal function, as acute illness or other factors leading to a patient’s ICU admission may affect baseline renal function. Finally, only internal validation was performed in this study, and external validation will be needed in the future to verify the applicability and robustness of the model.

## Conclusion

In the present study, we built and tested six clinical feature-based ML models, and it showed that the XGBoost model had an excellent performance for predicting AKI in critically ill patients. Further SHAP values and the LIME method indicated that SOFA score, body weight, mechanical ventilation and the SAPS II score were the marked contributors for the prediction of AKI. These findings would be helpful for clinical prediction and the improvement of risk stratification of AKI in critically ill patients.

### Electronic supplementary material

Below is the link to the electronic supplementary material.

Supplementary Material 1

## Data Availability

The datasets presented in the current study are available in the MIMIC IV database (https://physionet.org/content/mimiciv/1.0/).

## Abbreviations

AI: Artificial intelligence
AKI: Acute kidney injury
AUC: Area under the curve
eGFR: Estimated glomerular filtration rate
ICU: Intensive care unit
KNN: k nearest neighbour
LIME: Local Interpretable Model-Agnostic Explanation
MIMIC IV: Medical Information Mart Database for Intensive Care IV
ML: Machine learning
RF: Random forest
SAPS II: Simplified Acute Physiology Score II
Scr: Serum creatinine
SHAP: Shapley Additive ExPlanation
SOFA: Sequential Organ Failure Assessment
SVM: Support vector machine
XAI: Explainable AI
XGBoost: Extreme gradient boosting
