# Supplementary material for: Interpretable machine learning model for predicting acute kidney injury in critically ill patients
Source: BMC Med Inform Decis Mak. 2024 May 31;24:148. doi: 10.1186/s12911-024-02537-9 (PMC11140965; doi:10.1186/s12911-024-02537-9)
Supplement: Supplementary file 1 — Supplementary Material 1 [file 12911_2024_2537_MOESM1_ESM.docx]

**Table S1. Missing number (%) for variables**

| **Variables** | Missing number (%) |
| --- | --- |
| **Age** | 0(0) |
| **Sex, male** | 0(0) |
| **Weight** | 1082(2.0%) |
| **Ethnicity** | 0(0) |
| **Congestive heart failure** | 0(0) |
| **Peptic ulcer disease** | 0(0) |
| **Myocardial infarction** | 0(0) |
| **Peripheral vascular disease** | 0(0) |
| **Diabetes** | 0(0) |
| **Dementia** | 0(0) |
| **Chronic pulmonary disease** | 0(0) |
| **Rheumatic disease** | 0(0) |
| **Cerebrovascular disease** | 0(0) |
| **Cancer** | 0(0) |
| **Paraplegia** | 0(0) |
| **Liver disease** | 0(0) |
| **Renal disease** | 0(0) |
| **AIDS** | 0(0) |
| **Sepsis** | 0(0) |
| **Heart rate** | 84(0.1%) |
| **MAP** | 119(0.2%) |
| **Respiratory rate** | 138(0.2%) |
| **Body temperature** | 1733(3.2%) |
| **SpO_2_** | 130(0.2%) |
| **Hematocrit** | 442(0.8%) |
| **Hemoglobin** | 486(0.9%) |
| **Platelets** | 479(0.9%) |
| **WBC** | 478(0.9%) |
| **BUN** | 409(0.7%) |
| **Anion gap** | 673(1.3%) |
| **INR** | 5281(9.9%) |
| **Serum Creatinine** | 406(0.7%) |
| **Serum glucose** | 803(1.5%) |
| **Serum calcium** | 5102(9.6%) |
| **Serum chloride** | 607(1.1%) |
| **Bicarbonate** | 619(1.2%) |
| **Serum potassium** | 640(1.2%) |
| **Serum sodium** | 612(1.2%) |
| **PT** | 5280(9.9%) |
| **PTT** | 5572(10.5%) |
| **eGFR** | 0(0) |
| **Dialysis** | 0(0) |
| **Vasopressors use** | 0(0) |
| **Mechanical ventilation** | 0(0) |
| **SOFA score** | 0(0) |
| **SAPS II score** | 0(0) |

Abbreviations: AIDS: acquired immune deficiency syndrome, MAP: mean arterial pressure, SpO_2_: oxygen saturation, WBC: white blood cell, BUN: blood urea nitrogen, INR: international normalized ratio, PT: prothrombin time, PTT: partial thromboplastin time, eGFR: estimated glomerular filtration rate, SOFA: sequential organ failure assessment, SAPS II: Simplified Acute Physiology Score II.

**Table S2. Comparison of baseline characteristics between the training and test sets**

| **Variables** | **Total**  **(n =53150)** | **Training set**  **(n =42520)** | **Test set**  **(n =10630)** | **P value** |
| --- | --- | --- | --- | --- |
| **Age (years)** | 66.76 [54.49, 78.24] | 66.73 [54.51, 78.21] | 66.89 [54.42, 78.46] | 0.940 |
| **Sex, male, n (%)** | 29797 (56.1) | 23811 (56.0) | 5986 (56.3) | 0.568 |
| **Weight (kg)** | 78.45 [65.90, 93.10] | 78.50 [65.90, 93.10] | 78.20 [65.80, 93.00] | 0.425 |
| **Ethnicity, n (%)** |  |  |  | 0.413 |
| White | 35668 (67.1) | 28484 (67.0) | 7184 (67.6) |  |
| Black | 4874 (9.2) | 3898 (9.2) | 976 (9.2) |  |
| Other | 12608 (23.7) | 10138 (23.8) | 2470 (23.2) |  |
| **Congestive heart failure, n (%)** | 12622 (23.7) | 10057 (23.7) | 2565 (24.1) | 0.307 |
| **Peptic ulcer disease, n (%)** | 1457 (2.7) | 1166 (2.7) | 291 (2.7) | 1 |
| **Myocardial infarction, n (%)** | 8531 (16.1) | 6770 (15.9) | 1761 (16.6) | 0.109 |
| **Peripheral vascular disease, n (%)** | 5820 (11.0) | 4658 (11.0) | 1162 (10.9) | 0.958 |
| **Diabetes, n (%)** | 14613 (27.5) | 11649 (27.4) | 2964 (27.9) | 0.321 |
| **Dementia, n (%)** | 1930 (3.6) | 1570 (3.7) | 360 (3.4) | 0.139 |
| **Chronic pulmonary disease, n (%)** | 12398 (23.3) | 9907 (23.3) | 2491 (23.4) | 0.780 |
| **Rheumatic disease, n (%)** | 1717 (3.2) | 1364 (3.2) | 353 (3.3) | 0.577 |
| **Cerebrovascular disease, n (%)** | 8539 (16.1) | 6764 (15.9) | 1775 (16.7) | 0.049 |
| **Cancer, n (%)** | 7723 (14.5) | 6152 (14.5) | 1571 (14.8) | 0.425 |
| **Paraplegia, n (%)** | 2748 (5.2) | 2164 (5.1) | 584 (5.5) | 0.097 |
| **Liver disease, n (%)** | 5766 (10.8) | 4612 (10.8) | 1154 (10.9) | 0.992 |
| **Renal disease, n (%)** | 9386 (17.7) | 7441 (17.5) | 1945 (18.3) | 0.056 |
| **AIDS, n (%)** | 284 (0.5) | 232 (0.5) | 52 (0.5) | 0.522 |
| **Sepsis, n (%)** | 23901 (45.0) | 19164 (45.1) | 4737 (44.6) | 0.352 |
| **Heart rate (beats/minute)** | 82.83 [73.25, 94.16] | 82.81 [73.25, 94.13] | 82.96 [73.26, 94.25] | 0.390 |
| **MAP (mmHg)** | 77.52 [71.27, 85.61] | 77.53 [71.27, 85.58] | 77.50 [71.26, 85.82] | 0.843 |
| **Respiratory rate (beats/minute)** | 18.38 [16.39, 20.96] | 18.36 [16.39, 20.95] | 18.44 [16.41, 21.03] | 0.196 |
| **Body temperature (°C)** | 36.81 [36.59, 37.07] | 36.81 [36.59, 37.07] | 36.81 [36.58, 37.07] | 0.511 |
| **SpO_2_ (%)** | 97.07 [95.69, 98.36] | 97.07 [95.69, 98.35] | 97.07 [95.71, 98.36] | 0.656 |
| **Hematocrit (%)** | 35.20 [31.00, 39.60] | 35.20 [31.00, 39.60] | 35.20 [31.00, 39.60] | 0.327 |
| **Hemoglobin (g/dL)** | 11.70 [10.20, 13.20] | 11.70 [10.20, 13.20] | 11.60 [10.10, 13.20] | 0.484 |
| **Platelets (K/uL)** | 210.00 [158.00, 275.00] | 210.00 [158.00, 275.00] | 211.00 [159.00, 274.00] | 0.986 |
| **WBC (K/uL)** | 12.30 [8.80, 16.70] | 12.30 [8.80, 16.70] | 12.20 [8.80, 16.70] | 0.509 |
| **BUN (mg/dL)** | 19.00 [14.00, 30.00] | 19.00 [14.00, 30.00] | 19.00 [14.00, 30.00] | 0.249 |
| **Anion gap (mEq/L)** | 15.00 [13.00, 18.00] | 15.00 [13.00, 18.00] | 15.00 [13.00, 18.00] | 0.027 |
| **INR** | 1.30 [1.10, 1.50] | 1.30 [1.10, 1.50] | 1.30 [1.10, 1.50] | 0.788 |
| **Serum creatinine (mg/dL)** | 1.00 [0.80, 1.40] | 1.00 [0.80, 1.40] | 1.00 [0.80, 1.50] | 0.070 |
| **Serum glucose (mg/dL)** | 137.00 [113.00, 178.00] | 137.00 [113.00, 178.00] | 137.00 [113.00, 177.00] | 0.906 |
| **Serum calcium (mg/dL)** | 8.60 [8.10, 9.00] | 8.60 [8.10, 9.00] | 8.60 [8.10, 9.00] | 0.460 |
| **Serum chloride (mEq/l)** | 106.00 [102.00, 109.00] | 106.00 [102.00, 109.00] | 106.00 [102.00, 109.00] | 0.222 |
| **Bicarbonate (mmol/L)** | 24.00 [22.00, 27.00] | 24.00 [22.00, 27.00] | 24.00 [22.00, 27.00] | 0.937 |
| **BUN (mg/dL)** | 19.00 [14.00, 30.00] | 19.00 [14.00, 30.00] | 19.00 [14.00, 30.00] | 0.249 |
| **Serum potassium (mEq/L)** | 4.40 [4.00, 4.80] | 4.40 [4.00, 4.80] | 4.40 [4.00, 4.80] | 0.744 |
| **Serum sodium (mEq/L)** | 140.00 [137.00, 142.00] | 140.00 [137.00, 142.00] | 140.00 [137.00, 142.00] | 0.833 |
| **PT (s)** | 14.00 [12.40, 16.60] | 14.00 [12.40, 16.60] | 14.00 [12.40, 16.60] | 0.769 |
| **PTT (s)** | 31.30 [27.50, 40.00] | 31.30 [27.50, 39.90] | 31.40 [27.60, 40.10] | 0.154 |
| **eGFR, ml/min/1.73 m^2^** | 1.01 [0.81, 1.06] | 1.01 [0.81, 1.06] | 1.01 [0.81, 1.06] | 0.693 |
| **Dialysis, n (%)** | 1668 (3.1) | 1331 (3.1) | 337 (3.2) | 0.857 |
| **Vasopressors use, n (%)** | 2004 (3.8) | 1592 (3.7) | 412 (3.9) | 0.542 |
| **Mechanical ventilation, n (%)** | 38366 (72.2) | 30722 (72.3) | 7644 (71.9) | 0.487 |
| **SOFA** **score** | 4.00 [2.00, 6.00] | 4.00 [2.00, 6.00] | 4.00 [2.00, 6.00] | 0.880 |
| **SAPS II** **score** | 33.00 [25.00, 42.00] | 33.00 [25.00, 42.00] | 33.00 [25.00, 42.00] | 0.654 |

Abbreviations: AIDS: acquired immune deficiency syndrome, MAP: mean arterial pressure, SpO2: oxygen saturation, WBC: white blood cell, BUN: blood urea nitrogen, INR: international normalized ratio, PT: prothrombin time, PTT: partial thromboplastin time, eGFR: estimated glomerular filtration rate, SOFA: sequential organ failure assessment, SAPS II: Simplified Acute Physiology Score II.


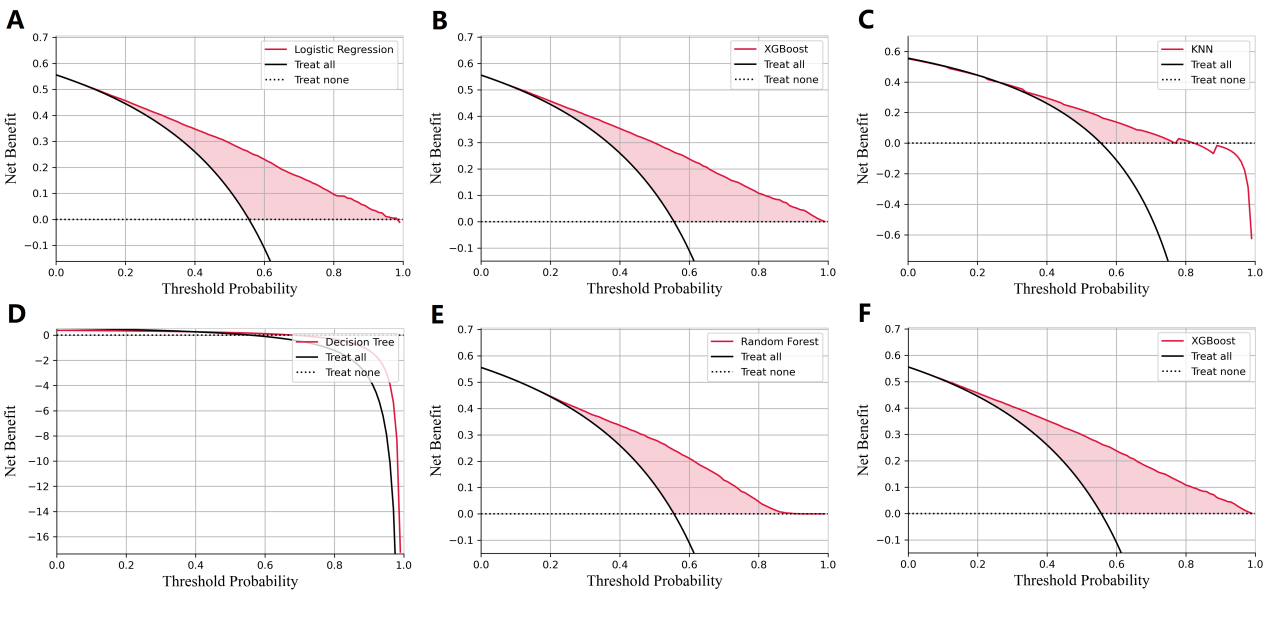


**Figure S1 The decision curve analysis curves for the six models**

(A) Logistic regression. (B) SVM. (C) KNN. (D) Decision tree. (E) Random forest. (F) XGBoost.

Abbreviations: SVM: support vector machine, KNN: k-Nearest neighbor, XGBoost: Extreme Gradient Boosting.


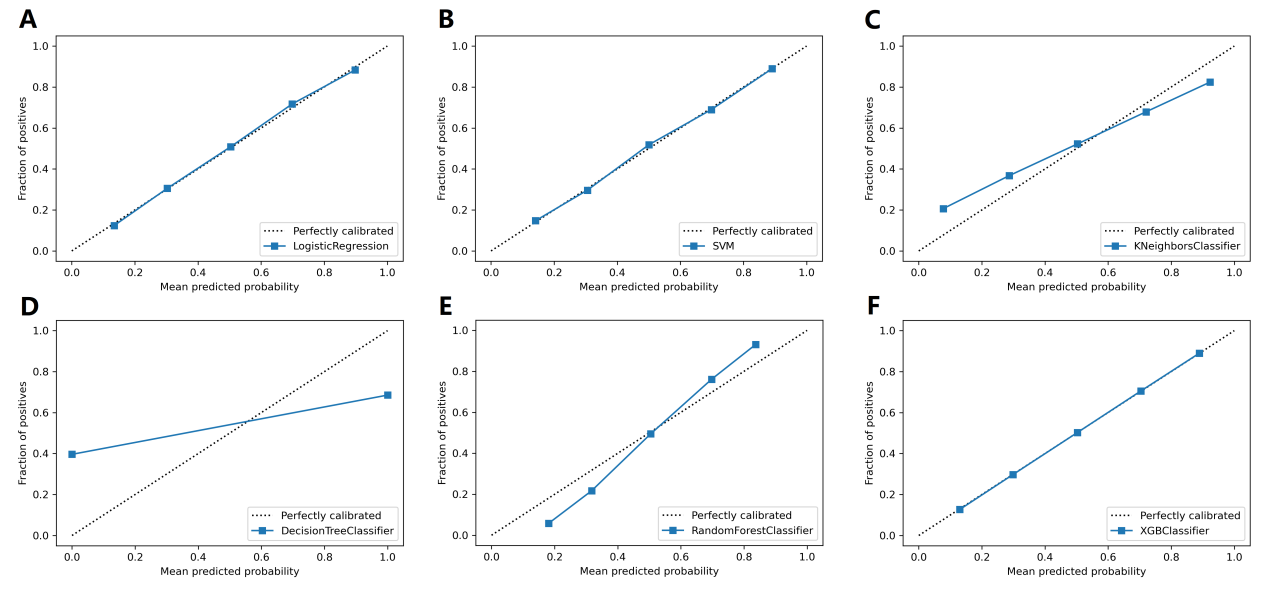


**Figure S2 The calibration** **curves for the six models**

(A) Logistic regression. (B) SVM. (C) KNN. (D) Decision tree. (E) Random forest. (F) XGBoost.

Abbreviations: SVM: support vector machine, KNN: k-Nearest neighbor, XGBoost: Extreme Gradient Boosting.
